# About-Weekly Pattern in the Dynamic Complexity of a Healthy Subject’s Cellular Immune Activity: A Biopsychosocial Analysis

**DOI:** 10.3389/fpsyt.2022.799214

**Published:** 2022-06-20

**Authors:** Lennart Seizer, Germaine Cornélissen-Guillaume, Günter K. Schiepek, Emil Chamson, Harald R. Bliem, Christian Schubert

**Affiliations:** ^1^Department of Psychiatry, Psychotherapy, Psychosomatics and Medical Psychology, Medical University of Innsbruck, Innsbruck, Austria; ^2^Institute of Psychology, University of Innsbruck, Innsbruck, Austria; ^3^Department of Integrative Biology and Physiology, Halberg Chronobiology Center, University of Minnesota, Minneapolis, MN, United States; ^4^Institute of Synergetics and Psychotherapy Research, Paracelsus Medical University, Salzburg, Austria; ^5^University Hospital of Psychiatry, Psychotherapy and Psychosomatics, Paracelsus Medical University, Salzburg, Austria; ^6^Department of Translation Studies, University of Innsbruck, Innsbruck, Austria

**Keywords:** neopterin, time series, dynamic complexity, non-linear, bioperiodicity, circaseptan, integrative single-case study, psychoneuroimmunology

## Abstract

In a previous integrative single-case study, we collected biological, psychological and social time-series data on a 25-year-old healthy woman over the course of 126 12-h intervals (63 days) and used urinary neopterin as an indicator of cellular immune activity [Schubert et al. 2012 (1)]. The present re-evaluation introduced Dynamic Complexity (DC) as an additional non-linear and non-stationary measure to further investigate the subject’s biopsychosocial dynamics during the study. The new time series dealing with urinary neopterin complexity revealed a cyclic, circaseptan (about-weekly) repeating pattern (6.59 days). The only weekly reoccurring events over the course of the study that were associated with this immunological pattern were the in-depth interviews with the subject (mean distance between interviews: 6.5 days). Superposed epoch analysis (SEA) revealed a U-shaped relation between neopterin complexity and interviews, with a decrease in neopterin complexity before and during interviews and an increase after interviews. Furthermore, the complexity scores for irritation, anxiousness/depressiveness and mental activity were positively correlated with neopterin complexity. The results suggest that the interviews, which had been found to be related to the subject’s need for educational and/or social accomplishment, were marked by stress (decrease in psycho-immunological flexibility and adaptability), which was then relieved after the interviews (increase in psycho-immunological flexibility and adaptability). It appears that the subject’s cellular immune activity, as indicated by neopterin complexity, functionally mirrored the emotional meaning she ascribed to the in-depth interviews. This re-evaluation is in line with the view that biopsychosocial research requires multimodal analysis of single cases based on qualitative (e.g., in-depth interviews) and quantitative (e.g., time series analysis) data under conditions of “life as it is lived”.

## Introduction

The systemic perspective of the biopsychosocial model (2, 3) assumes that nature is a hierarchically arranged continuum, with higher-order entities (e.g., society, family, and organism) being superordinate to those downstream (e.g., tissues, cells, and genes). Within this hierarchy of biological, psychological and social systems, complex dynamic interdependencies exist – e.g., top-down/bottom-up regulatory feedback circuits – that shape human experience and can be responsible for an individual’s health and disease (4). Moreover, from a biosemiotic perspective, living systems such as human beings are essentially driven by sign relations and their signification in life processes (5). In this view, the personal meaning of an incident (e.g., a stressor) is determined by both the context of the incident and the individual interpreting the incident. This interpretation process, in turn, is informed by a person’s current and past experience. In terms of meaning complexity, therefore, health and disease are highly dependent on subjective – and often subconscious – factors (6).

A central methodological challenge in biopsychosocial research is how to investigate such complex issues involving biological, psychological and social systems under conditions of high ecological validity (7). To study dynamic interdependencies, data are usually gathered in time series consisting of repeated measurements of variables. The ensuing statistical analysis needs to deal with linear as well as non-linear time series characteristics. Furthermore, the proper analysis of personally meaningful incidents requires qualitative methods (e.g., in-depth interviews, hermeneutic analysis, and consensus rating) to explore how subjective meaning emerges from the experience of an incident and how this meaning is communicated between subject and researcher (8).

In integrative single-case studies, we combine these qualitative and quantitative research aspects under conditions of “life as it is lived.” The study design requires that subjects collect their entire urine in 12-h intervals (biological), fill out questionnaires in the morning and evening (psychological) and participate in weekly interviews to identify the previous week’s emotionally meaningful daily incidents (social). In one integrative single-case study (1) on a healthy 25-year-old female subject who collected biopsychosocial data over the course of 63 days, i.e., 126 12-h intervals, the use of linear time series analysis (i.e., ARIMA modeling, cross-correlational analysis) led to new findings in stress research. One of these findings was that urinary neopterin concentrations [a cellular immune parameter (9, 10)] showed cyclic or biphasic response patterns lasting up to 60 h after the occurrence of emotionally meaningful everyday incidents. This suggests that the emotional processing of incidents takes days and is accompanied by a psycho-immunological regulatory feedback mechanism (1, 11, 12).

This finding is in line with the fact that in real-life, no physiological variables result in perfect constancy or periodicity, but rather are blurred by fluctuations around an underlying process. Such irregularities can be seen as noise, referring to unexplained variability from random influences, such as ever-changing environmental factors that force the organism to adapt, or as chaos resulting from a deterministic system with non-linear cause-effect relations (13). The cyclic psychobiological response patterns found in the original study (1) may indeed be indications of such dynamics. Consequently, for a further understanding of the subject’s biopsychosocial dynamics during the study period, an expansion of the applied statistical method is necessary from linear to non-linear.

Thus, the current re-evaluation of the original data set (1) used a dynamic complexity measure (14) to further analyze the connection between the psychosocial incidents identified as meaningful to the subject and her cellular immune functioning, as indicated by neopterin. Specifically, this re-evaluation dealt with the following questions: (1) Are patterns present in the new urinary neopterin complexity time series? (2) Are such neopterin complexity patterns temporally and, moreover, functionally related to the occurrence of emotionally meaningful psychosocial incidents?

## Methods

### Subject Description

The subject was a 25-year-old woman (height: 1.80 m; weight: 74 kg). She was a non-smoker and did not use oral contraceptives or other regular medications. Her parents broke up shortly after her birth. During her childhood, she was raised in large part by her grandparents and her aunt and saw her mother (45 years old) only on weekends. She had face-to-face contact with her biological father only once about 1 year before study start. At the time of the study, the subject was single and living in a studio apartment in Innsbruck, Austria. She was studying biology, working part-time at the university (10 h per week) and taking teacher-training courses (6–10 h per week). The subject saw her family every 3 weeks and her best friend every 2–3 days. She reported on four other close friends. Interviews conducted prior to study start and during the two-month study period followed by hermeneutic analysis and rating revealed that the subject’s need for educational and/or social accomplishment was a central personal theme (1).

### Study Design

Shortly before the start of the study, the subject was given thorough physical and psychiatric examinations and a psychological evaluation [“Check-up für Normalpersonen”, The Life Event and Difficulty Schedule (LEDS)]. During the study period, in which biological, psychological and social time-series data were gathered, she was examined weekly to monitor general health.

Biological time series: The subject collected her entire urine for a period of 63 days in 12-h intervals, lasting from approx. 8 a.m. to approx. 8 p.m. (day) and from approx. 8 p.m. to approx. 8 a.m. (night), leading to a total of 126 measurements. Upon collection, Na-Metabisulfite and Na-EDTA were added to the canister to prevent urine sedimentation and oxidation. After each 12-h collection interval, the urine samples were frozen by the subject at −20°C. Weekly, the subject brought the samples to the laboratory where they were stored at −70°C. Within 3 months following collection, urinary neopterin values were determined using high pressure liquid chromatography (HPLC; Model LC 550; Varian Associates, Palo Alto, CA, United States) (9), normalized against urinary creatinine (HPLC) and expressed as μmol neopterin per mol creatinine. Urinary neopterin mirrors the general biosynthesis of neopterin in the body well because it is not biologically active, does not bind to receptors and undergoes rapid renal clearance (10).

Psychological time series: Twice each day, at the end of each 12-h interval, i.e., at approx. 8 a.m. and 8 p.m., the subject filled out a set of questionnaires included in the Daily Inventory of Activity, Routine and Illness (DIARI) (11). These questionnaires concerned the preceding 12 h and consisted of the following: (1) “Eigenschaftswörterliste 60-S” (EWL 60-S) (15), which measures emotional states in six categories [mental energy levels, general lethargy, extraversion/introversion (introversion values are converted into extraversion values by reversing the sign), wellbeing, irritation, anxiousness/depressiveness] using a total of 60 adjectives, each of which is assessed on a four-point Likert scale. (2) Questions dealing with physical activity, alcohol and coffee consumption, drug use/medication, sleep, menstrual cycle, body temperature, potential signs of a cold, flu, etc., (3) Notes on all positive and negative psychosocial incidents, even minor ones.

Social time series: Once a week, the Incidents and Hassles Inventory (IHI) (11), a semi-structured in-depth interview lasting approx. 1 h, was conducted with the subject to evaluate the past week’s emotionally meaningful incidents. At the end of the study, these incidents were reviewed in detail with regard to time of occurrence, extent of anticipation, meaningful people involved, memories evoked, etc.

The subject of this study gave informed consent in written form to her participation and to the publication of data. The Ethics Committee of the Medical Faculty of the University of Innsbruck approved the design. The subject received 11,150 Austrian Schillings (approx. 800 euros) for her participation. For further details on the study design, see (1).

### Dynamic Complexity

According to Goldberger and colleagues, “complexity arises from the interaction of a myriad of structural units and regulatory feedback loops that operate over a wide range of temporal and spatial scales, enabling the organism to adapt to the stresses of everyday life” (16). There are several methods to evaluate dynamic complexity, e.g., pointwise correlation dimension, local largest Lyapunov exponent and entropy rates. These methods are preferable to variance measures, for example, because they not only contain information on varying degrees of fluctuation but are also sensitive to the organization, shape and (in)stability of a process. However, they usually require long sets of data points with high temporal resolution. To avoid this issue, a specific measure of dynamic complexity was developed that is applicable to short and coarse-grained real-world time series, without further statistical or parametric assumptions (14, 17). This measure, known simply as Dynamic Complexity (DC), was applied in this re-evaluation.

DC is the product of multiplying a fluctuation measure (F) by a distribution measure (D) and is applied to discrete time series data with given data ranges (xmin, xmax) and constant discrete time intervals between the data points. F is sensitive to the amplitudes and frequencies of a time signal, and D scans the scattering of values or system states occurring within the range of possible values or system states. F and D are normalized to be 0 ≤ X ≤ 1; the precise formulas can be seen in (14). In order to identify non-stationarity, DC is calculated within a data window moving over the time series. Because the time series we use in this study were collected in 12-h intervals, we apply a window width of seven measurement points, corresponding to 3.5 days. This calculation results in a new time series that reflects the dynamic complexity of the original data.

### Statistical Analyses

All statistical analyses were conducted using R 4.0.5 (18). To identify dominant frequencies and repeating patterns in the DC time series of neopterin, autocorrelation functions (ACF) and spectral analysis were applied after first-order differentiation (19). To evaluate the impact of discrete events on the neopterin complexity time series, a superposed epoch analysis (SEA) (20, 21) was computed. To do so, vectors of data points around each discrete event were combined into a composite matrix, with the width of the matrix indicating the number of data points around the event and the length of the matrix the number of events. In this study, a period of 3 days (six 12-h intervals, i.e., lags) before and after each of the discrete events was chosen (total window of 13 12-h intervals). The complexity scores of each vector (matrix rows) were normalized by expressing them as changes in percentage of their arithmetic mean to allow better comparability of different temporal periods. Differences between lags (matrix columns) were then evaluated using polynomial regression analysis to investigate potential patterns of neopterin complexity before and after events. To evaluate the associations between urinary neopterin and EWL scales (both raw data and dynamic complexities), Pearson product-moment correlations were calculated. Prior to correlation analyses, time series were stationarized through differentiation when necessary (augmented Dickey-Fuller test). In all analyses, statistical significance was considered at *p* < 0.05.

## Results

**FIGURE 1:**
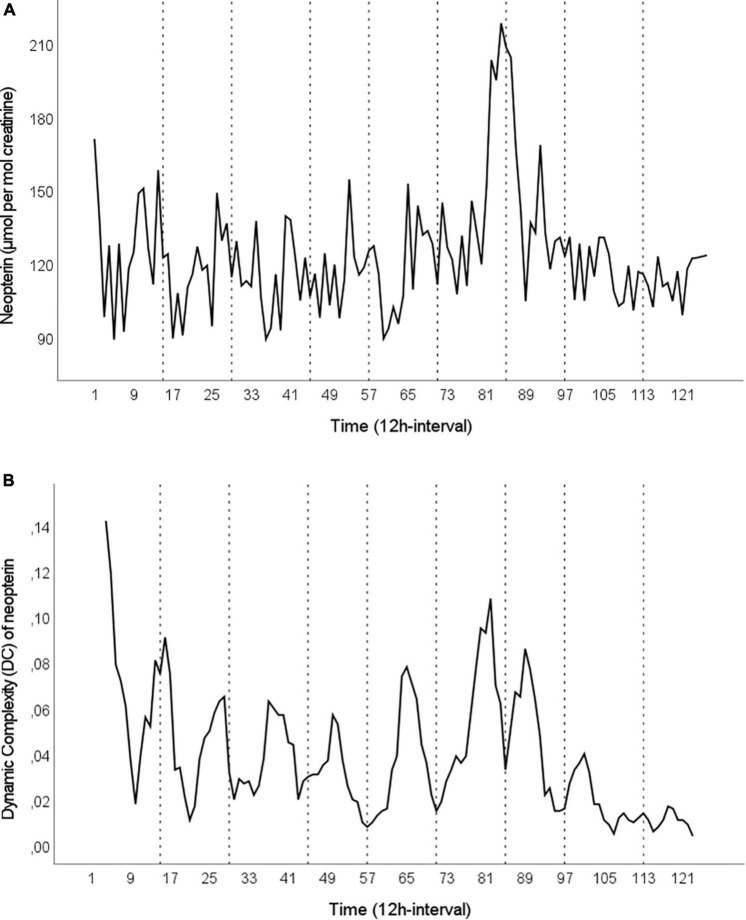
**(A)** Time series of urinary neopterin in μmol per mol creatinine over a period of 63 days in 12-h intervals (*n* = 126). The data were collected in daytime intervals (8 a.m.–8 p.m., odd numbers) and nighttime intervals (8 p.m.–8 a.m., even numbers). **(B)** The Dynamic Complexity (DC) of neopterin over the course of the study in moving windows of seven 12-h intervals (*n* = 120). In both figures, dotted vertical lines represent the time points of interviews.

Figure 1 shows the raw time series of urinary neopterin concentrations in μmol per mol creatinine (A) and the time series of DC scores for urinary neopterin (B). Urinary neopterin per creatinine concentrations had a mean of 123.63 ± 24.05 (89.01–218.27) μmol per mol creatinine, with DC scores averaging 0.039 ± 0.026 (0.004–0.142).

**FIGURE 2:**
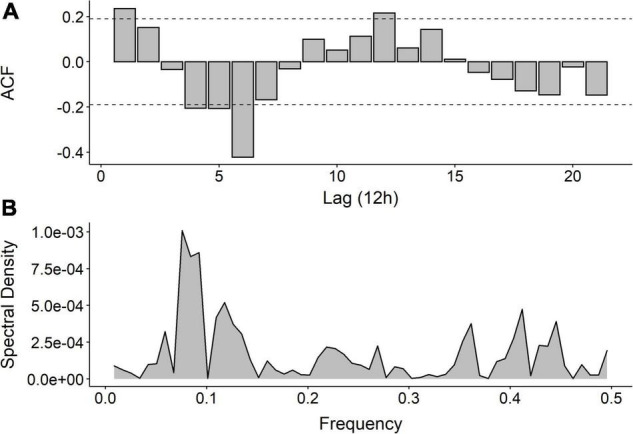
**(A)** Autocorrelation function (ACF) of Dynamic Complexity (DC) of urinary neopterin per creatinine up to 21 lags. Coefficients (bars) reaching the upper or lower confidence limits (dotted lines) are significant at *p* < 0.05. **(B)** Periodogram of DC of urinary neopterin per creatinine.

The ACF of the urinary neopterin complexity time series showed significant positive autocorrelations at lag 1 (*r* = 0.235, *p* = 0.005) and at lag 12 (*r* = 0.216, *p* = 0.009) and a significant negative autocorrelation at lag 6 (*r* = −0.421, *p* < 0.001) (Figure 2A). The spectral density of urinary neopterin complexity peaked at a frequency of 0.076. This indicates a repeating pattern of 13.18 time units (12 h), i.e., 6.59 days, which represents a circaseptan or about-weekly rhythm (Figure 2B). However, this about-weekly rhythm in neopterin complexity neither corresponded to a certain calendar schedule, such as weekends, nor to DIARI variables such as sleep duration per night, subjective sleep quality, caffeine or alcohol consumption, medication use, body temperature, menstrual cycle or physical activity (data not shown). This is in line with interview analyses showing no social or work-related weekly rhythm that remained constant throughout the entire study period. Specifically, the first half of the study took place during December and January when the university’s winter break was held. During this time, the subject stayed with her family and did not go to courses or to work. In the second half of the study, the subject resumed her normal university/work routine with regular weekly obligations.

**FIGURE 3:**
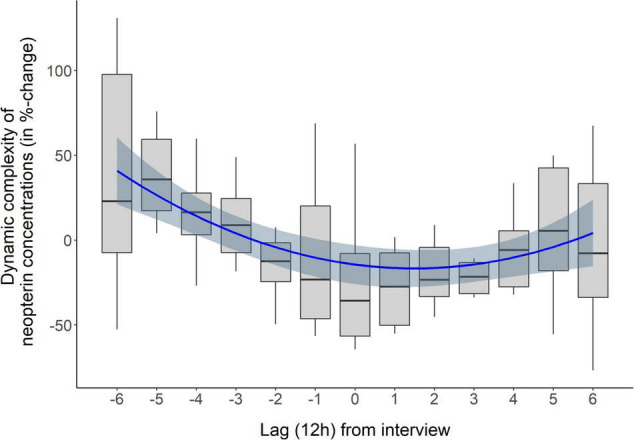
Superposed epoch analysis (SEA) of in-depth interviews (*n* = 8) and Dynamic Complexity (DC) of urinary neopterin levels, expressed as change in percentage of the arithmetic mean, during a window of ± 6 lags (12 h), i.e., 3 days before and after interviews. Boxplots represent median (horizontal lines), quartiles (boxes), and range (vertical lines) of neopterin DC values at given lags. Interviews took place at lag 0. A polynomial curve with a linear and a quadratic estimator was fitted to the data (blue line with blue shaded standard error).

The only about-weekly reoccurring events over the course of the study period were the in-depth interviews with the subject (mean distance between interviews: 6.5 days). Therefore, we sought to determine whether the interviews and associated emotions might have driven the repeating pattern in neopterin complexity. Figure 1B shows that most of the interviews correspond to lows in neopterin complexity. An SEA with a temporal window of ± 6 lags, i.e., 3 days before and after an interview (lag 0), was performed to assess the impact of interviews on neopterin complexity. The results (percentage changes of arithmetic mean) are presented in Figure 3 and show a U-shaped pattern, with a decrease in neopterin complexity before and during interviews and an increase after interviews. A polynomial regression was fitted to the SEA data, with a linear (β_1_ = −17.43, *p* = 0.004) and quadratic term (β_2_ = 1.03, *p* = 0.001). This model explained 17% of the variance in neopterin complexity change [*R*^2^ = 0.17, *F*(2,101) = 10.01, *p* < 0.001].

Next, we performed Pearson product-moment correlations between urinary neopterin and the six EWL scales (both raw data and dynamic complexities) to test whether changes in emotional states correlated with changes in urinary neopterin. Raw urinary neopterin concentrations correlated only with extraversion/introversion (*r* = −0.211, *p* = 0.018). Urinary neopterin complexity, however, correlated significantly with the complexity scores of irritation (*r* = +0.243, *p* = 0.007), mental activity (*r* = +0.262, *p* = 0.005) and anxiousness/depressiveness (*r* = +0.24, *p* = 0.008) but not with the complexity scores of general lethargy (*r* = +0.01, *p* = 0.9), extraversion/introversion (*r* = +0.05, *p* = 0.61), or wellbeing (*r* = −0.07, *p* = 0.44).

## Discussion

In this investigation, we re-evaluated the psychosocial and immunological data from a previous integrative single-case study (1) and considered DC as an additional non-linear and non-stationary measure to further investigate the subject’s biopsychosocial dynamics during the course of the study. The central finding of this re-evaluation was that the newly calculated neopterin complexity time series, which was based on the 12-h to 12-h variability of neopterin concentration in the urine samples, revealed a repeating cyclic pattern of circaseptan or about-weekly length (6.59 days). This pattern appeared to be functionally related to the in-depth interviews (mean distance between interviews: 6.5 days) conducted with the subject during the study period.

Before discussing these findings in more detail, one might wonder whether there are alternative explanations for the about-weekly neopterin complexity pattern found in this study. In this context, the “life as it is lived” approach of the integrative single case design yielded a large amount of time series data from diverse biopsychosocial entities which can now be used to identify any factors potentially influencing the findings of this re-evaluation.

Specifically, an external stimulus could have produced the changes in DC of neopterin (22), but the regularity of the pattern makes it unlikely to have been triggered by erratic events such as infections or irregularly occurring psychosocial incidents (1). Another explanation for the about-weekly pattern in neopterin complexity might have been social or cultural rhythms, which give rise to e.g., workdays and weekends that may be associated with various behavior and activity patterns in everyday life (23). In the present re-evaluation, however, the about-weekly rhythm in neopterin complexity did not correspond to a certain calendar schedule such as weekends or to any other variables covered by the DIARI. This was in line with interview analyses showing no social or work-related weekly rhythms that remained constant throughout the entire study period.

The about-weekly neopterin complexity pattern might alternatively be an inherent biorhythm synchronized to a natural phenomenon. Several free-running about-weekly physiological rhythms have been identified by previous research (24–26), although little is known about their origin or biological implications. For example, such rhythms might be related to persistent cosmic phenomena, such as about-weekly geomagnetic cycles (27–30) or about-weekly phases of the lunar cycle (31, 32). In this re-evaluation, it cannot be ruled out that such an inherent about-weekly biorhythm might have interfered with the discovered immunological pattern.

The only weekly reoccurring events that were temporally related to the about-weekly neopterin complexity pattern were the in-depth interviews of the study (mean distance between interviews: 6.5 days). In order to understand this relationship on a functional level, the special character of the interviews and their meaning to the subject need to be outlined. While the interviews were not psychotherapeutic interventions *per se*, they did include therapeutic elements such as the discussion of emotionally positive or negative incidents, the reflection of the subject’s own thoughts, feelings, actions and coping strategies, and the potential disclosure of suppressed psychological material (see excerpts from interviews 2 and 8 in Table 1). This nature of the interviews appears to have touched the specific personal theme of the subject – namely, “educational and/or social accomplishment,” which was discovered in the first evaluation of this study. This theme concerns the subject’s need to elevate her self-worth through successful completion of various socially approved activities, e.g., university exams, meetings at work, academic presentation and, as the original study found, the in-depth interviews during the study. Because of their challenging character, incidents related to this theme were, as the interviews showed, often precipitated by negative emotions (“nervous”, “under stress”, “uncomfortable”, “agitated”, “taxing”) and followed by positive emotions (“self-content”, “fun”, “moved”, “pleasant”, “satisfied”, “reaffirmed”, “high spirits”, “relieved”, “energized”, “happy”) (1).

**TABLE 1:** Excerpts from the weekly in-depth interviews referring to the previous week’s interview.

Interview no.	Interviewer	Subject
1	-	-
2	How did the interview with you go last week?	Not so great. Since I was pretty depressed from the week with all the problems and then I had to rehash everything, tell everything again, I wasn’t quite sure afterward whether I was the patient now or what was going on, it confused me a bit, it was quite strange. I would have felt more comfortable if I had been able to tell nice things rather than all the bad things again that have been on my mind all week.
3	Was there anything about the interview last week?	It was quite long. The device didn’t work. (The subject refers to the voice recording device.)
4	How was the interview last week? Did you find anything upsetting or uncomfortable in any way?	I can only remember that I actually left in quite high spirits.
5	How was the interview last week?	I can’t say anything good or bad.
6	Was there anything about the interview last week?	I can’t remember, no.
7	Was there anything about the interview itself?	No.
8	How was the interview last week – was there anything special?	There was something, yes. You somehow asked if there was anything that could be done about that, with Dad at home and all that, yes… It’s a topic that I don’t like to think about anymore or I don’t like to deal with because it’s already been talked through so much, and I went through it a lot for many years with [name of friend], and somehow I’ve recognized that I can’t do anything about it. Probably by now, I don’t even want to waste the energy to somehow do something, that’s why I don’t like to talk about it, that was really it.
9	Was there anything about the interview last week?	I can’t remember, no.

*Column 1 indicates which interview the quotes are taken from. Columns 2 and 3 indicate the questions from the interviewer and the subject’s answers (translated from German). No information is given for the first interview because there was no interview beforehand. Also, no information is given for the ninth and final interview because no further interview followed. In the first interview, the voice recording device did not work; therefore, the main points from the first interview were reiterated during the second interview.*

We believe that the connection to the subject’s personal theme may have rendered the in-depth interviews of the study meaningful and that this connection might now help to explain how the in-depth interviews and the urinary neopterin complexity time series were functionally related. The U-shaped pattern in the SEA shows that the closer the interviews came, the more the subject’s neopterin complexity decreased, whereas after interviews, neopterin complexity began to increase (see Figure 3). As little is known about the relation between psychological factors and measures of immune complexity, it may be helpful to look at other physiological systems to interpret our findings. In this regard, much is already known about the influence of psychological factors on cardiac complexity (33). Specifically, under normal conditions, the natural cardiac pacemaker, which is responsible for heart rate (HR), fluctuates between a set of metastable states or attractors and can switch at any time from one state to another quickly, thereby adapting to ever-changing internal and external conditions (34, 35). Evidence suggests that when HR complexity is higher, the pacemaker shows a higher degree of freedom and a greater range of possible adaptive responses (36). Interestingly, studies have shown that under acute stress, HR complexity decreases (33, 37). This change toward more stable and periodic HR behavior under stress may be associated with stronger regularity, decoupling of multimodal integrated networks and deactivation of control-loops within the cardiovascular system. Thus, stress-related reduction in HR complexity may represent a lower adaptability and fitness of the cardiac pacemaker and a functional restriction of the participating cardiovascular elements (38–40).

Similarly, the immune system is a complex dynamic multiscale system consisting of molecular (e.g., genes, proteins), cellular, and organismal networks that act in an organized manner to foster effective functioning (41, 42). As with the cardiac system, immune activity does not work independently from other internal and external systems but rather in a complex supersystem comprising social, psychological, neurological, endocrinological, and immunological entities: the socio-psycho-neuro-endocrino-immunological network (8, 43). Neopterin-releasing cells (macrophages, monocytes, dendritic cells, endothelial cells, and fibroblasts) (44) play an important role in this network and appear to be, like many other immune components, characterized by non-linear behaviors arising from dynamic, feedback-regulated interactions (41, 42). In this regard, neopterin, whose production is dependent upon interferon-γ (IFN-γ) stimulation, might be an indicator of cellular [T-helper type 1 (Th1)] immune complexity – just like HR is an indicator of cardiac complexity (9, 10).

With regard to the connection between psychosocial and immune factors, evidence from psychoneuroimmunology (PNI) clearly shows that psychosocial interventions can modulate immune system activity (8, 45, 46). As to the PNI findings of this re-evaluation, the similar about-weekly rhythms of the in-depth interviews and neopterin complexity may be an indicator of synchronized or coupled dynamics between both system levels. Stress-related brain mechanisms may play an important role as a quasi-causal link between cognition, emotion and behavior at the psychological level and the dynamics of the immune system. Synchronization may result from intersystemic connections or from external pacemakers, whereas coupling implies direct effects of one system on the other (17). The in-depth interviews may be related to the immune dynamics shown in this re-evaluation in that the interviews simply mirror the mental (psychosocial) experiences of the subject, or play the role of a pacemaker (47, 48). From this perspective, the subject’s personal theme, “educational and/or social accomplishment,” and the emotions connected to the interviews might have acted as a control parameter of the synchronized stress system dynamics (order parameters), as evidenced by neopterin complexity.

As to the specific functional connection between in-depth interviews and urinary neopterin complexity found in this re-evaluation, we assume that the decreasing neopterin complexity days before and during interviews was related to stress that rendered the psycho-immunological system less flexible and, thus, less adaptable. Thereafter, however, the subject was relieved, ultimately leading to a restoration of the flexibility/adaptability of the emotional and immune systems (see in Figure 3 the slight increase in neopterin complexity after interviews) (49). In line with this interpretation, neopterin complexity was positively correlated with DC scores in irritation, anxiousness/depressiveness, and mental activity. A shift in the DC of neopterin may therefore be understood not only as a change in cellular (Th1) immune activity but also as a change of state in the overall psycho-immunological system (50–52). However, while most of the interviews fit the local low points in the time series of neopterin complexity (see Figure 1B), the first and, to some extent, the second interview break ranks and coincide with local high points in complexity. Since this study was performed in a real-life setting, incidents or influences other than the interviews may have been responsible for these local high points. An alternative explanation is that the first interviews were different from the following ones because they were conducted at the beginning of the study when the whole procedure was new and the subject had no previous experience with the interview format (see Table 1, interview 2). For the subsequent interviews, the subject was able to anticipate the procedure and content (see section “Methods”). Interestingly, the periodic pattern in neopterin complexity became less pronounced toward the end of the study period (see Figure 1B), which might be explained by weaker psychophysiological responses, either because the subject habituated to the in-depth interviews or she was glad to be approaching the end of the study.

In sum, the findings of this re-evaluation suggest that the about-weekly pattern in the subject’s urinary neopterin complexity was an expression of a whole-person adaptation toward the emotionally meaningful in-depth interviews during the study. Specifically, our findings indicate that the subject’s personal theme, “educational and/or social accomplishment,” contributed to the meaningfulness of the interviews, with the subject’s urinary neopterin time series immunologically reflecting her engaged participation in the study. Findings further indicate functional relations between the interviews and the subject’s psycho-immunological responses: The interviews were initially marked by stress, accompanied by decreases in psycho-immunological flexibility and adaptability, and then followed by relief, accompanied by increases in psycho-immunological flexibility and adaptability. Such results on complex psycho-immunological functioning go far beyond the findings from our first evaluation (1).

One limitation of this study is that we cannot rule out that the about-weekly pattern in urinary neopterin complexity is an intrinsic one. Specifically, there was no longer period either preceding or following the study in which the subject collected her urine for neopterin determination but did not participate in weekly in-depth interviews. Moreover, only little data was gathered on how the subject experienced the weekly interviews. More information would have allowed for a much better understanding of the relation between interviews and neopterin complexity. Furthermore, only urinary neopterin was evaluated in terms of DC. The evaluation of a broader range of immunological and endocrinological markers could have led to a better description of the physiological processes involved. Also, the results of this re-evaluation do not permit generalization since they refer to only one subject; replication in further studies is required. As biopsychosocial findings from various studies cannot be generalized well *via* conventional nomothetic-deductive means, generalization could instead be based on an ideographic-inductive approach applying systematic statistical comparison (53, 54).

In conclusion, biopsychosocial complexity research in life science requires time series analysis, with frequent, equidistant measures of variables over a long study period. In addition, the interpretation of such data involves careful analysis of contextual factors including subjective meaning. In this context, subjective meaning is a function of personal experience, which is connected to the (subconscious) personal themes and conflicts an individual has acquired throughout life (8). To this end, the integrative single-case approach uses extensive single-case analyses under conditions of “life as it is lived,” integrating time and meaning in order to access the full richness of a person’s complex biopsychosocial reality.

## Data Availability Statement

The raw data supporting the conclusions of this article will be made available by the authors, without undue reservation.

## Ethics Statement

The studies involving human participants were reviewed and approved by the Ethics Committee of the Medical Faculty of the University of Innsbruck. The patients/participants provided their written informed consent to participate in this study and to the publication of data.

## Author Contributions

CS performed the study. LS, CS, GC-G, and GKS analyzed the data. LS, CS, GC-G, GKS, HB, and EC wrote the manuscript. All authors contributed to the article and approved the submitted version.

## Conflict of Interest

The authors declare that the research was conducted in the absence of any commercial or financial relationships that could be construed as a potential conflict of interest.

## Publisher’s Note

All claims expressed in this article are solely those of the authors and do not necessarily represent those of their affiliated organizations, or those of the publisher, the editors and the reviewers. Any product that may be evaluated in this article, or claim that may be made by its manufacturer, is not guaranteed or endorsed by the publisher.
